# Cortical Pain Processing in the Rat Anterior Cingulate Cortex and Primary Somatosensory Cortex

**DOI:** 10.3389/fncel.2019.00165

**Published:** 2019-04-24

**Authors:** Zhengdong Xiao, Erik Martinez, Prathamesh M. Kulkarni, Qiaosheng Zhang, Qianning Hou, David Rosenberg, Robert Talay, Leor Shalot, Haocheng Zhou, Jing Wang, Zhe Sage Chen

**Affiliations:** ^1^Department of Instrument Science and Technology, Zhejiang University, Hangzhou, China; ^2^Department of Psychiatry, New York University School of Medicine, New York, NY, United States; ^3^Department of Anesthesiology, Perioperative Care and Pain Medicine, New York University School of Medicine, New York, NY, United States; ^4^Department of Biophysics, University of Science and Technology of China, Hefei, China; ^5^New York University School of Medicine, New York, NY, United States; ^6^Department of Neuroscience and Physiology, New York University School of Medicine, New York, NY, United States; ^7^Neuroscience Institute, New York University School of Medicine, New York, NY, United States

**Keywords:** evoked pain, spontaneous pain, anterior cingulate cortex (ACC), primary somatosensory cortex (S1), phase-amplitude coupling (PAC)

## Abstract

Pain is a complex multidimensional experience encompassing sensory-discriminative, affective-motivational and cognitive-emotional components mediated by different neural mechanisms. Investigations of neurophysiological signals from simultaneous recordings of two or more cortical circuits may reveal important circuit mechanisms on cortical pain processing. The anterior cingulate cortex (ACC) and primary somatosensory cortex (S1) represent two most important cortical circuits related to sensory and affective processing of pain. Here, we recorded *in vivo* extracellular activity of the ACC and S1 simultaneously from male adult Sprague-Dale rats (*n* = 5), while repetitive noxious laser stimulations were delivered to animalÕs hindpaw during pain experiments. We identified spontaneous pain-like events based on stereotyped pain behaviors in rats. We further conducted systematic analyses of spike and local field potential (LFP) recordings from both ACC and S1 during evoked and spontaneous pain episodes. From LFP recordings, we found stronger phase-amplitude coupling (theta phase vs. gamma amplitude) in the S1 than the ACC (*n* = 10 sessions), in both evoked (*p* = 0.058) and spontaneous pain-like behaviors (*p* = 0.017, paired signed rank test). In addition, pain-modulated ACC and S1 neuronal firing correlated with the amplitude of stimulus-induced event-related potentials (ERPs) during evoked pain episodes. We further designed statistical and machine learning methods to detect pain signals by integrating ACC and S1 ensemble spikes and LFPs. Together, these results reveal differential coding roles between the ACC and S1 in cortical pain processing, as well as point to distinct neural mechanisms between evoked and putative spontaneous pain at both LFP and cellular levels.

## Introduction

Pain is a complex sensory experience involving multidimensional components, encoded by distributed cortical pain circuits. For example, the primary somatosensory cortex (S1) is known to represent the sensory-discriminative component of pain (Vierck et al., 2013), whereas the anterior cingulate cortex (ACC) is known to represent the affective-motivational component of pain (Bushnell et al., 2013). Human neuroimaging experiments have suggested that many other neocortical regions, such as the insular, secondary somatosensory cortex, prefrontal cortex, and orbitofrontal cortex, also play important roles in pain processing (Davis et al., 2017). With regards to the duration of pain experiences, pain is often classified as acute and chronic pain. Stimulus-evoked pain is induced by a noxious stimulus, whereas spontaneous pain is detached from an overt external stimulus. It is known that repeated noxious stimulations can elicit spontaneous pain behaviors (Bennett, 2012); however, identification of spontaneous pain remains challenging in animal studies (Tappe-Theodor and Kuner, 2014).

At the single cell level, ACC neurons are known to encode the affective component of pain experiences, and chronic pain may enhance the aversive responses of ACC neurons (Zhang et al., 2017; Zhou et al., 2018). At the mesoscopic and macroscopic levels, intracortical local field potential (LFP) signals provide important physiological signatures for characterizing pain at a fine timescale comparable to that of single neuronal activity (Ploner et al., 2017; Peng et al., 2018; Ploner and May, 2018). The EEG-based *phase-locked* event-related potentials (ERPs) have been suggested for identifying biomarkers for pain (Pinheiro et al., 2016). The ERP amplitude reflects the degree of synchrony within local neuronal populations. A power increase or decrease is referred to as *non-phase-locked* event-related synchronization or desynchronization (ERS or ERD), respectively (Bressler, 2002). ERPs are referred to as “evoked potentials” when occurring soon after a stimulus, and spontaneous in the period without any stimulus presentation. If ERPs are not directly evoked by overt stimuli, they are sometimes called as “induced potentials.” While ERP was primarily used in EEG analyses, here we adapted this concept for LFP recordings. In pain experiments, ERPs are often temporally associated with stereotyped pain behaviors (such as the paw withdrawal and licking in rodents) (Cheppudira, 2006; Kawasaki et al., 2012; Whittaker and Howarth, 2014; Murai et al., 2016). Since identifying spontaneous pain in rodent studies is difficult due to the lack of pain report, ERPs may be viewed as a proxy measure of pain (Davis et al., 2017). Throughout the paper, we use the term ERP interchangeably for both evoked pain and spontaneous pain-like episodes, which are referred to as pain-evoked potentials and pain-induced potentials, respectively.

In this report, we focused the investigation on simultaneous ACC and S1 recordings, and examined the differences between evoked pain and spontaneous pain-like episodes. We combined animal behavior, neurophysiology (spikes and LFP), and machine learning to examine the neural codes during identified pain episodes in freely behaving rats. While single neuronal spikes present precise timing information related to sensory coding or representation, it imposes a technical challenge to obtain stable ensembles in chronic recordings over days. In contrast, LFPs represent the aggregate subthreshold activity of neurons in a local localized area, and are relatively stable over time, thereby providing a reliable macroscopic readout from local microcircuits related to pain processing.

## Materials and Methods

### Animals and Protocols

All experimental studies were performed in accordance with the National Institutes of Health (NIH) *Guide for the Care and Use of Laboratory Animals* to ensure minimal animal use and discomfort, and were approved by the New York University School of Medicine (NYUSOM) Institutional Animal Care and Use Committee (IACUC). Male adult Sprague-Dale rats (250–300 g, Taconic Farms, Albany, NY) were used in our current study and kept at the new Science Building at NYUSOM, with controlled humidity, temperature and 12-h (6:30 a.m.–6:30 p.m.) light-dark cycle. Food and water were available *ad libitum*. Animals were given on average 10 days to adjust to the new environment before the initiation of experiments.

**Figure 1:**
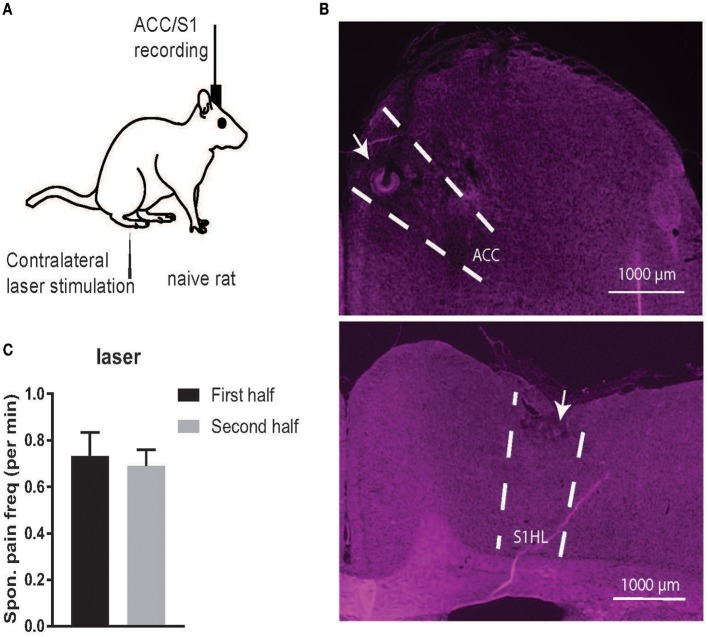
**(A)** Schematic diagrams of noxious stimulation and recording on naive rats. **(B)** Histology of recording areas in the rat ACC and S1. **(C)** Comparison of the frequency (per minute) of spontaneous pain-like episodes between the first and second half of recording sessions. There was no statistical difference between the first and second half (*p* > 0.05, paired *t*-test).

Thermal pain stimuli were used for rats freely exploring in a plastic chamber of size 38 × 20 × 25 cm^3^ on top of a mesh table. A blue (473 nm diode-pumped solid-state) laser with varying laser intensities was delivered to the animal's hindpaw (Figure 1A). The laser stimulation was delivered in repeated trials (25–40) during 30–45 min. During experiments, two video cameras (120 frame per second) were used to continuously monitor the animal's behavior. The rat's evoked pain behavior was characterized by its latency to paw withdrawal (Cheppudira, 2006; Deuis et al., 2017).

### Identification of Spontaneous Pain-Like Events Based on Stereotyped Behaviors

Repeated noxious stimulus stimulations to the rat hindpaw could induce spontaneous pain-like behaviors. Between the inter-trial intervals of laser stimulations, we examined the animal's behavior to search for putative spontaneous pain episodes. Specifically, we categorized the spontaneous pain-like behavior into several subtypes: (i) twitch—short and sudden jerking movement that was not associated with locomotion or a pain stimulus; (ii) lifting/flicking—the lift of the foot would not involve movement of the whole leg or bending of the knee; (iii) paw withdrawal and paw licking—taking the foot up and into the body with bending of the knee, which is often accompanied by shaking the foot. Lick is characterized as lifting the foot off of the grating and licking it. The licks mirrored the licking involved immediately after a pain stimulus. None of these behaviors were associated with locomotion or a pain stimulus.

Due to the lack of ground truth, we referred to those events as spontaneous pain-like episodes. Such behaviors could also indicate pain anticipation due to repeated stimulations (Barrot, 2012; Urien et al., 2018).

### Electrode Implant and Electrophysiology

We used silicon probes (Buzsaki32, NeuroNexus) with 3D printed drive to record multi-channel (up to 64 channels) neural activities from the rat ACC and S1 areas simultaneously, at the contralateral side of the paw that received noxious laser stimulation. For surgery, rats were anesthetized with isoflurane (1.5–2%). The skull was exposed and a 3 mm-diameter hole was drilled above the target region. The coordinates for the ACC and S1 implants were: ACC: AP 2.7, ML 1.4–2.0, DV 2.0, with an angle of 20° toward the middle line; S1: AP −1.5, ML 2.5–3.2, DV 1.5. The drive as secured to the skull screws with dental cement.

Using a Plexon (Dallas, TX) data acquisition system, we recorded *in vivo* neurophysiological signals at a 40 kHz sampling rate, and band-pass filtered (0.3 Hz–7.5 kHz). Spikes were thresholded from the high-passed (> 300 Hz) raw signals, and the subsequent band-pass filtered (1–100 Hz) signals produced LFPs. Detected spikes were further sorted offline using commercial software (Offline Sorter, Plexon).

A total of 15 animals were used in the current study: 10 naive rats were used for behavioral analysis only, and another 5 naive rats were used for both behavioral and physiological analyses (Table 1). In rats #11-13, we recorded LFP signals from the ACC and S1 simultaneously using silicon probes (Figure 1B). In rats #14–15, we obtained both LFP recordings and well-isolated units from the ACC and S1. In total, we have analyzed 81 ACC units and 41 S1 units from these two rats.

**Table 1:** Summary of experimental data.

**Animal**	**Stimulus**	**Behavior**	**LFP**	**# Unit analysis**	**# Sessions**
		**analysis**	**analysis**	**(ACC + S1)**	**per rat**
naive rats #1–5	None	Yes	n/a	n/a	1
naive rats #6–10	blue laser	Yes	n/a	n/a	1
naive rat #11	blue laser	Yes	Yes	0 + 0	2
naive rat #12	blue laser	Yes	Yes	0 + 0	2
naive rat #13	blue laser	Yes	Yes	0 + 0	3
naive rat #14	blue laser	Yes	Yes	33 + 5	2
naive rat #15	blue laser	Yes	Yes	48 + 36	3

### Identification of Pain-Modulated Units

Triggered on the stimulus onset, we computed the peri-stimulus time histogram (PSTH) of each neuron (50 ms bin size). From the PSTH, we identified the positively (or negatively) pain-modulated units from the S1 and ACC (Chen et al., 2017; Zhang et al., 2017), which showed significantly increased (or decreased) firing rates compared to the baseline (5 s window before the stimulus onset).

At a finer timescale, we also computed the ERP peak-triggered PSTH for each neuron using a 10 ms bin size. The PSTH was further smoothed using a Gaussian kernel with a bandwidth of 20 ms.

### Identification of ERPs

A cortical ERP reflects the coordinated behavior of a large number of neurons in relation to an external or internal event. Traditional ERP analysis is based on trial averaging (Garcia et al., 2002). In contrast, all studies reported here were based on single-trial analyses. From LFP recordings, we identified the induced ERPs during evoked and spontaneous pain episodes. Since the ERP was associated with low-frequency activity, to remove the high-frequency noise, we further applied band-pass filtering (2–15 Hz) to the multi-channel LFP traces, followed by principal component analysis (PCA). We extracted the dominant principal component that produced an ERP waveform associated with the largest variance, which was used in the subsequent ERP analysis. The duration of the ERP waveform was 200–250 ms, and the signal-to-noise ratio (SNR) varied in each evoked pain or spontaneous pain-like episode. We used a conservative SNR criterion for the ERPs, and sought ERPs around the pain-like behaviors (within a window of [−5, 5] s centered at the event onset).

### Spectrum and Time-Frequency Analyses

The coherence measures the amplitude-amplitude coupling between two random signals across a wide range of frequencies. The spike-field coherence (SFC) measures phase synchronization between the LFP and spike times as a function of frequency. The coherence and SFC is scaled between 0 and 1. We assumed trial stationarity and derived trial-averaged coherence and SFC, as well as their jackknife error bars. In single-trial analyses, we computed the spectrogram or SFC in the time-frequency representation by using a moving window.

Multitapered spectral analyses for LFP spectrogram, LFP coherence and SFC were performed using the Chronux toolbox (chronux.org). Specifically, we chose a half-bandwidth parameter *W* such that the windowing functions were maximally concentrated within [−*W, W*]. We chose *W* > 1/*T* (where *T* denotes the duration) such that the Slepian taper functions were well concentrated in frequency and had bias reducing characteristics. In terms of Chronux function setup, we used the tapers setup [*TW, N*], where *TW* is the time-bandwidth product, and *N* = 2 × *TW*−1 is the number of tapers. Since the taper functions are mutually orthogonal, they give independent spectral estimates. In all time-frequency analyses, we used a moving window length of 0.5–1 s and a step size of 1 ms. In the LFP coherence and SFC analyses, a 2 s window was used across all pain episodes. We used *TW* = 5 for LFP spectrogram and coherence, and *TW* = 3 for SFC. From the spectrogram, we computed the Z-scored spectrogram, where the baseline was defined as the 5-s period before the noxious stimulus presentation.

### Cross-Frequency Phase-Amplitude Coupling

PAC was used to characterize the coupling between the low-frequency (theta) phase and high-frequency (gamma) amplitude of EEG recordings during nociception (Wang et al., 2011). We adapted the PAC analysis to LFP recordings during pain episodes. Specifically, we band-pass filtered LFP signals into proper frequency (theta or gamma) band and computed their Hilbert transform. From the derived complex-valued signals, we extracted the instantaneous theta phase and gamma amplitude (envelope), and further constructed the phase-amplitude histogram (18 bins within 0–360°). In light of the phase-amplitude distribution, we ran a parametric test based on the generalized linear model (GLM) to assess the PAC. A quantitative scalar statistic *r* = max{|1−*A*_*s*_./*A*_0_|} (where *A*_*s*_ and *A*_0_ denote the predicted amplitude vectors defined at the phase vector [0, 2π] using the spline model and the null model, respectively; ./ denotes the dot division operator in MATLAB) along with its confidence intervals was reported (Kramer and Eden, 2013).

### Detection of the Onset of Pain Signals

We have previously developed model-based methods for detecting the onset of acute pain signals based on the ACC and/or S1 neural ensemble spike activity (Chen et al., 2017; Hu et al., 2018; Xiao et al., 2018). Assuming that subjective pain signal was latent and evolved dynamically in time, we proposed a Poisson linear dynamical system (PLDS) to link the pain stimulus to neural spiking activity of a population of *C* Poisson-spiking neurons, as follows:

(1) $$\begin{array}{l} z_{t}={az}_{t-1}+\epsilon_{t} \end{array}$$

(2) $$\begin{array}{l} y_{t}\sim \text{Poisson}\left(\exp(cz_{t}+d)\Delta \right) \end{array}$$

where ${\text{y}}_{t}={\left[y_{1,t},\ldots ,y_{C,t}\right]}^{⊤}$ denotes a *C*-dimensional population vector, with each element consisting of the neuronal spike count within the time interval ((*t*−1)Δ, *tΔ*] (bin size Δ); the univariate (latent) variable *z*_*t*_ represents the latent common input that drives the neuronal population firing rate. The dynamics of the latent variable is governed by a first-order autoregressive (AR) model (0 < |*a*| < 1) driven by a zero-mean Gaussian noise process $\epsilon_{t}\in N\left(0,\sigma_{\epsilon }^{2}\right)$. The parameters *c* and *d* are unconstrained. During evoked pain episodes, we used an expectation-maximization (EM) algorithm to estimate the unknown state variables *z*_1:*T*_ and all unknown parameters {*a, c, d*, σ_ϵ_} from the spike count observations **y**_1:*T*_ in a single trial.

During spontaneous pain episodes, we assumed that the model parameters were identical to those derived from a previous evoked pain episode, and ran a recursive (forward) filtering algorithm to estimate the latent state variable ẑ_*t*|*t*_ (Hu et al., 2018)

$$\begin{array}{l} {ẑ}_{t|t-1}=a{ẑ}_{t-1|t-1}\\ Q_{t|t-1}=a^{2}Q_{t-1|t-1}+\sigma_{\epsilon }^{2}\\ {\hat{\text{y}}}_{t|t-1}=\exp\left(c{\hat{z}}_{t|t-1}+d\right)\Delta \\ Q_{t|t}^{-1}=Q_{t|t-1}^{-1}+c^{⊤}diag\left({\hat{\text{y}}}_{t|t-1}\right)c\\ {ẑ}_{t|t}={ẑ}_{t|t-1}+Q_{t|t}{\text{c}}^{⊤}\left({\text{y}}_{t}-{\hat{\text{y}}}_{t|t-1}\right) \end{array}$$

where *Q*_*t*|*t*_ = Var[ẑ_*t*|*t*_] denotes the filtered state variance. Furthermore, we computed the Z-score with respect to the pre-stimulus baseline: $\text{Z-score}=\frac{{ẑ}_{t}-\text{mean of }z_{\text{baseline}}}{\text{SD of }z_{\text{baseline}}}$ and converted it to probability:

(3) $$\begin{array}{l} P\left(\text{Z-score}>\theta_{0}\right)=1-\int_{-\infty }^{\theta_{0}}\frac{1}{\sqrt{2\pi }}e^{\frac{-u^{2}}{2}}du \end{array}$$

The criterion of Z-score change was determined by a statistical threshold θ_0_ depending on the significance level. We used θ_0_ = 1.65, which is associated with a *P*-value of 0.05. Finally, we identified the onset of pain signal when the Z-score crossed the significance threshold (Chen et al., 2017).

### LFP-Based Classification of Spontaneous Pain-Like Events

For each spontaneous pain-like episode, we constructed a window of [−5, 5] s centered around the behavior onset. Based on the simultaneous LFP recordings from the rat ACC and S1, we computed the LFP power features at five different frequency bands: theta (4–8 Hz), alpha (8–12 Hz), beta (13–30 Hz), lower gamma (30–50 Hz), high gamma (50–80 Hz), for both pre-behavior ([−5, 0] s) and post-behavior ([0, 5] s) periods separately. In total, we used 5 × 2 × 2 = 20 power features. Each power feature was preprocessed with zero mean and unit variance. At each recording session, we also selected the pain-free control baseline prior to the first pain stimulus presentation. In total, we constructed 252 spontaneous pain episodes (positive examples) and 252 negative control examples for training and testing (rats #12-15, *n* = 10 sessions).

We trained these features with a two-class support vector machine (SVM) classifier (Scholkopf and Smola, 2001). The SVM is a discriminative supervised learning model that constructs the classification boundary by a separating hyperplane with maximum margin. Specifically, the SVM maps the input **x** into a high-dimensional feature space and maximizes the margin from the hyperplane to the origin. The nonlinear decision function can be written as follows

$$\begin{array}{l} y\left({\text{x}}_{i}\right)=\overset{n}{\underset{i=1}{\sum }}\alpha_{i}K\left(\text{x},{\text{x}}_{i}\right)+b \end{array}$$

where *y*_*i*_ ∈ {−1, +1} denote the class label for the training sample **x**_*i*_ (some of which associated with nonzero α_*i*_ are called support vectors), *b* denotes the bias, and *K*(·, ·) denotes the kernel function. We used a polynomial kernel and trained the nonlinear SVM with a sequential minimal optimization algorithm (MATLAB Machine Learning Toolbox: “fitcsvm” function). The decoding accuracy was assessed by 5-fold cross-validation. The chance level of classification accuracy is 50%. We also assessed the sensitivity and specificity of SVM classifier by reporting the AUROC (area under the curve of receiver operating characteristic). The chance level of AUROC is 0.5 (Zhang et al., 2017; Dale et al., 2018).

We have tested both linear and Gaussian kernels in SVM. The nonlinear SVM produced a slightly better classification accuracy, but the feature weights derived from the linear SVM would yield informative assessment or interpretation of each feature.

### Statistical Tests

We conducted a paired or unpaired *t*-test provided that the data normality was satisfied; otherwise, we used a nonparametric signed-rank test or rank-sum test.

## Results

### Frequency of Spontaneous Pain-Like Behaviors

In animal studies, pain cannot be measured directly; therefore, pain can only be inferred from stereotyped “pain-like” behaviors (Deuis et al., 2017). Evoked pain episodes are uniquely associated with the noxious stimulus presentation and quantitative pain behaviors (e.g., paw withdrawal or lifting) (Cheppudira, 2006); whereas spontaneous pain-like behaviors often involve frequent aberrant movement such as flinching, shaking, paw lifting and paw licking (Kawasaki et al., 2012; Whittaker and Howarth, 2014; Murai et al., 2016).

When splitting the time of each recording session in half, the total spontaneous pain-like behaviors occurred in a similar frequency (per minute) in time during the course of a recording session (Figure 1C). As a control, we also examined naive rats (rats #1–5) in a completely pain-free condition (Day 1, without presenting any noxious stimulus) based on the same behavior criterion. The number of identified spontaneous pain-like behaviors was zero in the control condition.

In addition, we computed the number and latency statistics of identified spontaneous pain-like events. The median number of spontaneous pain-like events between two evoked pain episodes was 1 (min: 0; max: 5); and the median latency from the previous evoked pain episode was 26.9 s (min: 5.1 s; max: 4.5 min).

### ERPs During Evoked and Spontaneous Pain-Like Behaviors

**Figure 2:**
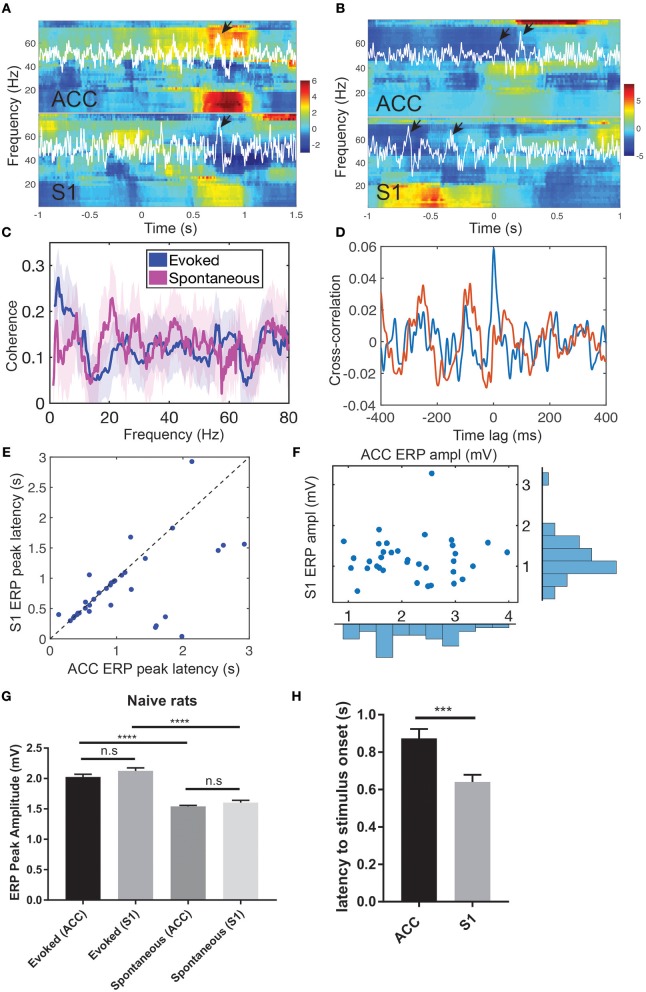
Pain-induced ERPs of the rat ACC and S1. **(A)** Simultaneous recordings of LFPs from the ACC and S1 induced by evoked pain and their Z-scored spectrograms. White LFP traces were the dominant principal component extracted from multi-channel LFP signals. Arrows indicate the identified ERPs. Time 0 indicates the stimulus onset. **(B)** Simultaneous recordings of LFPs from the ACC and S1 induced by spontaneous pain and their Z-scored spectrograms. Arrows indicate the identified ERPs. Using the ACC LFP as a reference, time 0 indicates the onset of identified ERP in the ACC. **(C)** LFP coherence between the ACC and S1 during evoked pain (red) and spontaneous pain-like (blue) episodes. Shaded area denotes the confidence intervals. **(D)** Cross-correlation between the band-pass filtered LFP (4–80 Hz) ACC and S1 from two representative single trials during evoked pain episodes (rat #15). Note that the red trial had a peak at the zero lag, whereas the blue trial showed rhythmic activity at a theta cycle (~200 ms). **(E)** Latency of ERP peak to the stimulus onset in evoked pain: ACC vs. S1 (*n* = 40 trials). **(F)** Peak-to-trough amplitude of ACC vs. S1 ERP in evoked pain. **(G)** Comparison of ACC and ERP peak amplitudes between evoked and spontaneous pain in naive rats. n.s., nonsignificant; ^****^p < 0.0001, unpaired *t*-test. **(H)** The latency from the ACC and S1 ERP peak amplitude to the laser stimulus onset during evoked pain (rats #12–15). Error bar shows SEM. ^***^p < 0.001, unpaired *t*-test.

During evoked pain episodes ([0, 5] s from the stimulus onset 0), ERPs occurred either in the ACC or S1 separately, or in both regions simultaneously; in the latter case, their ERP latencies differed (Figure 2A). The mean ± SEM latency from the ERP peak amplitude to the laser stimulus onset was 0.875 ± 0.050 s in the ACC and 0.640 ± 0.038 s in the S1 (Figure 2H, *p* = 0.0002, rank-sum test). During spontaneous pain-like episodes ([−5, 5] s centered around the behavior onset), ERPs did not always occur synchronously (Figure 2B and Figure S1; ratio: 367/638 in ACC and 367/497 in S1), and they occurred more frequently in the ACC than in the S1 (63.8 ± 8.36 per session and a total of 638 within 10 sessions in ACC; 49.7 ± 8.17 per session and a total of 497 within 10 sessions in Figures S1C,D). In the spectrogram, pain-evoked ERPs were accompanied with an increased theta band (4-8 Hz) power—known as the theta-ERS (Figures 2A,B). The ERS was also visible in the gamma band (30–80 Hz). The gamma-ERS was mostly separated in the slow (30–50 Hz) and fast (>50 Hz) gamma bands.

During spontaneous pain-like episodes, ERPs tended not to occur together in the ACC and S1, and the gamma-ERS was more pronounced in the S1. In either of the two regions, we often observed the theta-ERS followed by the gamma-ERS (Figure 2B and Figures S1A,B,E,F). At the trial-average level, there was enhanced coherence in the theta band between the ACC and S1 during evoked pain episodes (Figure 2C), but not in spontaneous pain-like episodes (peak coherence evoked: 0.26 ± 0.08 vs. mean coherence spontaneous: 0.13 ± 0.08).

During evoked pain episodes, the cross-correlation of LFPs between the ACC and S1 varied between single trials—for instance, having a high peak at zero time lag in one trial, or having a rhythmic theta cycle in another trial (e.g., see Figure 2D). When ERPs occurred simultaneously in both the ACC and S1 regions, the S1 had shorter ERP latencies to the stimulus onset than the ACC (Figure 2E; *p* = 0.0113, paired *t*-test, rat #12), but had comparable ERP amplitudes (Figure 2F; *p* > 0.05, paired *t*-test). See Figure S2 for population statistics. As pain behavior was characterized by the paw withdrawal latency (to the stimulus onset at time 0), we correlated the paw withdrawal latency with the evoked ERP latency (Figure S3A) and the ERP amplitude (Figure S3B), respectively. We found strong positive (or negative) correlations (*p* < 10^−4^) between the paw withdrawal latency and ERP latency (or amplitude).

Furthermore, we compared the ERP peak amplitude between evoked pain and spontaneous pain-like episodes (Figure 2G). There was no statistical difference in evoked ERP amplitude between the ACC and S1 (*p* > 0.05, unpaired *t*-test). However, the ERP amplitude was significantly greater in evoked pain than spontaneous pain-like episodes, in both the ACC and S1 (*p* < 0.0001).

During spontaneous pain-like episodes, when ERPs occurred together (within a window of ±3 s) in two areas, the S1-ERP tended to appear earlier than the ACC-ERP. The averaged lag was 0.080 ± 0.054 s (*n* = 367, rats #12–15), which shared a similar latency trend as in evoked pain.

Put together, these results suggest that pain-induced ERPs correlate well with pain-like behaviors and can serve as a neural signature readout of evoked pain and spontaneous pain-like episodes. During evoked pain episodes, the ERPs in the ACC and S1 were comparable in latency and amplitude; whereas during spontaneous pain-like episodes, ERPs did not always appear together in the ACC and S1, and appeared more frequently in the ACC.

### Phase-Amplitude Coupling

**Figure 3:**
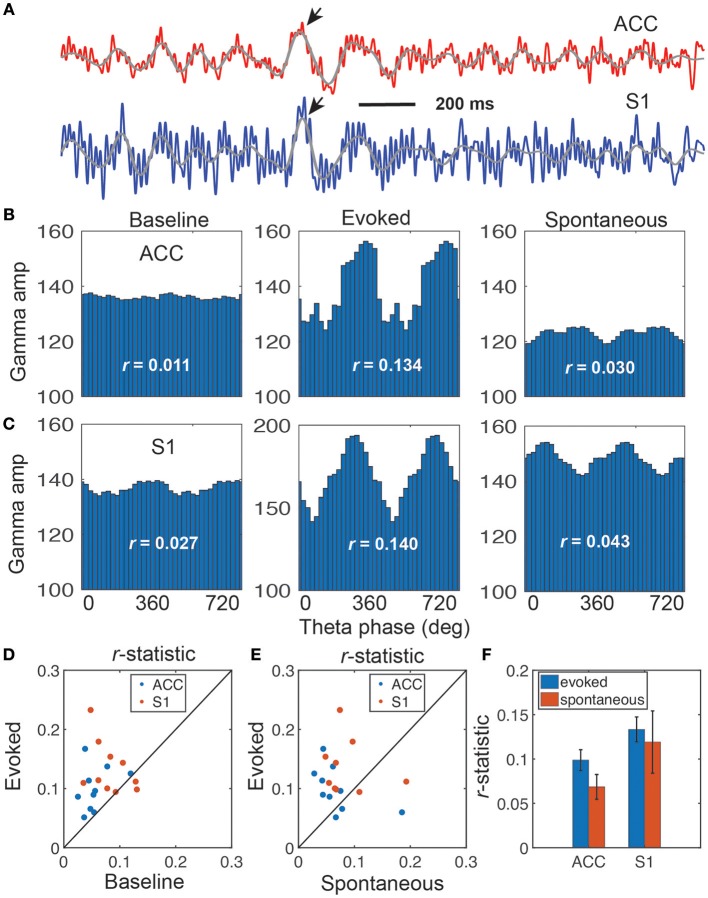
Illustration of coupling between the theta phase and gamma amplitude. **(A)** Representative LFP traces from the rat ACC (red) and S1 (blue), which shows strong coupling between the theta phase and gamma amplitude (rat #15). Two arrows indicate the identified ERPs, and two overlaid gray traces indicate the bandpass filtered (4–11 Hz) LFP traces. **(B,C)** Coupling of gamma amplitude and theta phase (0–720°) in the ACC **(B)** and S1 **(C)**: baseline (left), evoked pain (middle), and spontaneous pain (right). In each panel, the *r*-statistic is shown. **(D)** Scatter plot comparison of *r*-statistic (red: ACC, blue: S1) between evoked pain and baseline (*n* = 10 sessions, rats #12–15). Paired signed-rank test, *p* = 0.0013. **(E)** Scatter plot comparison of *r*-statistic (red: ACC, blue: S1) between evoked pain and spontaneous pain-like episodes (*n* = 10 sessions). Paired signed-rank test, *p* = 0.079. **(F)** Comparison of *r*-statistic in the S1 and ACC. Error bar denotes SEM (*n* = 10 sessions).

Phase-amplitude coupling (PAC) is useful to characterize nonlinear interactions between two different frequency oscillations (Canolty and Knight, 2010; Tort et al., 2010). Specifically, phase (theta) and amplitude (gamma) coupling has been reported in rat EEG recordings during acute pain experiments (Wang et al., 2011). We extended this analysis to the rat LFP recordings in ACC and S1 areas (Figure 3A). We found significant PAC in both ACC and S1 areas, and the strength of coupling was stronger in evoked pain than spontaneous pain-like episodes (Table 2 and Figures 3B,C). In addition, the gamma power showed a significant increase from baseline to evoked pain in both areas; however, during spontaneous pain-like episodes, there was an increase of gamma power in the S1, but not in the ACC (Figures 3B,C). This finding supports the notion that the S1 gamma-ERS is indicative of pain perception. In addition, the strength of PAC coupling was different between the ACC and S1, and varied between sub-gamma bands (Figure S4). The preferred phases of the ACC and S1 might differ between evoked pain and spontaneous pain-like episodes (Figure S5A), whereas the ACC and S1 also had different preferred theta phases (Figure S5B).

**Table 2:** Results of phase-amplitude coupling for the *r*-statistic (95% confidence intervals shown in bracket, and the greatest values in each region are shown in bold font).

**Rat**	**ACC**			**S1**		
	**Baseline**	**Evoked**	**Spontaneous**	**Baseline**	**Evoked**	**Spontaneous**
#12	0.030[0.026, 0.053]	0.084[0.072, 0.101]	0.056[0.047, 0.073]	0.028[0.023, 0.050]	0.154[0.127, 0.175]	0.075[0.060, 0.093]
#13	0.053[0.041, 0.077]	0.105[0.091, 0.132]	0.031[0.025, 0.044]	0.072[0.060, 0.099]	0.108[0.103, 0.134]	0.069[0.058, 0.085]
#14	0.042[0.030, 0.061]	0.064[0.052, 0.083]	0.050[0.043, 0.064]	0.057[0.046, 0.073]	0.083[0.075, 0.101]	0.065[0.051, 0.081]
#15	0.052[0.045, 0.081]	0.113[0.088, 0.145]	0.076[0.060, 0.096]	0.069[0.053, 0.102]	0.138[0.116, 0.176]	0.110[0.092, 0.154]

These PAC results were consistent across all rats (Figures 3D,E and Table 2). Overall, the strength of PAC in evoked pain was stronger than in baseline (*p* = 0.0013, paired signed-rank test; Figure 3D), and showed an increasing trend compared to spontaneous pain-like episodes (*p* = 0.079, Figure 3E). Combining all tested rats, we found a stronger coupling strength in the S1 than ACC, during both evoked pain (*p* = 0.058, paired signed-rank test) and spontaneous pain-like episodes (*p* = 0.017; Figure 3F).

### LFP Power

**Figure 4:**
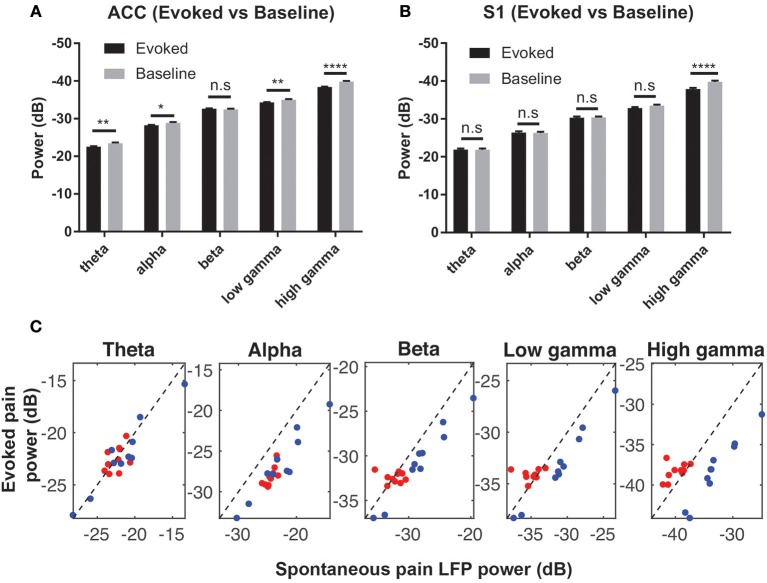
**(A)** Comparison of the mean LFP power between evoked pain and baseline at various frequency bands in the ACC **(A)** and S1 **(B)** (rats #12–15, *n* = 232 trials). Note that the y-axis shows the negative value in dB. ^*^p < 0.05; ^**^p < 0.01, ^****^p < 0.0001, paired *t*-test. **(C)** Mean LFP power comparison between the evoked and spontaneous pain-like episodes (ACC: red; S1: blue; rats #12–15, *n* = 10 sessions).

We computed the trial-averaged LFP power at the theta (4–8 Hz), alpha (8–12 Hz), beta (13–30 Hz), low-gamma (30–50 Hz) and high-gamma (50–80 Hz) frequency bands. There was an increase in high-gamma power from baseline ([−5, 0] s) to evoked pain ([0, 5] s, with 0 being the stimulus onset) in both the ACC and S1 (Figures 4A,B). During spontaneous pain-like episodes, we extracted the LFP signals within a window of [−5, 5] s centered around the event onset, and found a reduction in alpha and beta power for both regions. In contrast, gamma power reduced in the ACC but increased in the S1 during spontaneous pain-like episodes (Figure 4C).

### Spike-LFP Modulation

We further investigated the relationship between the pain-modulated ACC or S1 unit activity and the LFP amplitude. To do so, we varied the intensity of noxious stimulation across trials to produce a wider range of firing rate changes.

**Figure 5:**
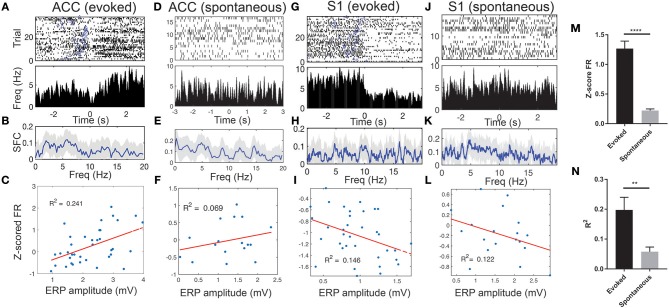
Spike phase locking of one representative ACC units and one representative S1 unit simultaneously recorded during evoked pain and spontaneous pain-like episodes (rat #15). **(A)** Spike raster and ERP-triggered peri-stimulus time histogram (PSTH). Bin size: 10 ms. Time 0 represents the peak of ERP. Blue triangle at each row indicates the onset of laser stimulation at each trial. Note that these two units decreased their firing rates around time 0. **(B)** Spike-field coherence (SFC). Shaded areas denote the jackknife error bar. Note that there was a peak in the theta frequency band. **(C)** Correlation between the Z-scored firing rate (FR) of pain-modulated units and the ERP peak-to-trough amplitude (*p* = 0.002, Pearson's correlation). **(D–F)** Similar to **A–C**, the same ACC unit during spontaneous pain-like episodes. **(G–L)** Similar to **A–F**, except for the S1 unit. In panels **F,I,L**, the *P*-values of Pearson's correlation are 0.307, 0.023, and 0.142, respectively. **(M)** Population statistics of Z-scored mean FR (in absolute value) of pain-modulated ACC and S1 units (*n* = 32) during evoked pain and spontaneous pain-like episodes. **(N)** Population statistics of *R*^2^ values (for regressing the Z-scored FR and the ERP amplitude). ^**^p < 0.01, ^****^p < 0.0001, unpaired *t*-test.

We observed a sharp change in the ERP peak-aligned ACC or S1 neuronal spike activity (Figures 5A,G), and this abrupt change became less pronounced when neuronal spike trains were aligned with the stimulus onset (Figure S6). Notably, these pain-modulated units showed a sharp reduction in firing rates around the ERP peaks. A close examination of the unit's PSTH revealed striking theta oscillations in spike activity (Figures S7A–C). During evoked pain episodes, we also observed enhanced trial-averaged SFC in the theta frequency band (Figure 5B; see Figure S7D for a single-trial analysis). Meanwhile, the SFC also varied between the evoked and spontaneous pain conditions (Figures 5B vs. 5E; Figures 5H vs. 5K). In addition, these pain-modulated ACC or S1 units showed a strong correlation with the evoked ERP amplitude, regardless of their response properties (Figures 5C,I).

During spontaneous pain-like episodes, pain-modulated ACC and S1 units showed reduced firing rate modulations (Figures 5D,J) and reduced modulations in relation to the ERP amplitudes (Figures 5F,L). However, the rhythmic theta spiking was still preserved (Figures S7E–H). Overall, the Z-score firing rates of pain-modulated ACC and S1 units and their modulation degree to ERP amplitudes were greater in evoked pain than spontaneous pain-like episodes (Figures 5M,N).

### Detection of Evoked Pain and Spontaneous Pain-Like Events

**Figure 6:**
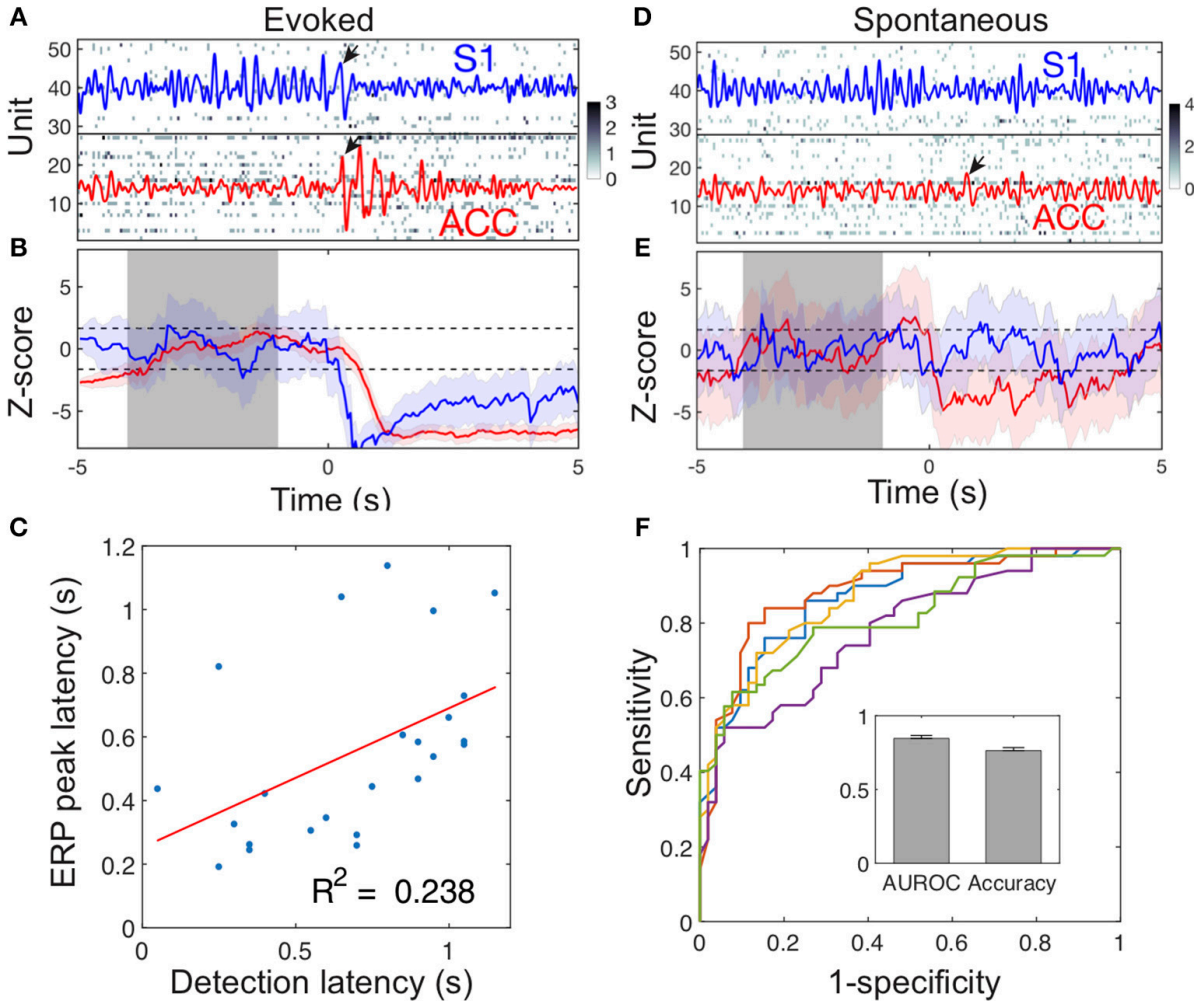
Illustration of detecting acute and spontaneous pain-like events. **(A)** The simultaneous ACC and S1 ensemble spike activity during a laser-evoked pain episode. Dark pixel represents high spike count. Time 0 denotes the onset of 250 mW laser stimulation. The ACC (red) and S1 (blue) LFPs were overlaid in the spike raster. Pain-induced ERPs are marked by arrows. In this example, the S1 ERP peak occurred 30 ms earlier than the ACC ERP peak. **(B)** The evoked pain event was detected based on our previously developed algorithm (Methods). Shaded periods of [−4, −1] s denote the baseline for computing the Z-score (ACC: red; S1: blue). When the upper or lower confidence interval of the Z-score was below or above the significance threshold (horizontal dash lines), the onset of pain signal was detected as a change point. In this example, the detected acute pain onset in the S1 was earlier than the onset in the ACC, whereas the S1 ERP peak latency was also earlier than the ACC ERP peak latency. **(C)** During evoked pain episodes, the ensemble spike-based acute pain detection latency to the stimulus onset positively correlated with the ERP peak latency (*n* = 24 trials; *p* = 0.0156, Pearson's correlation). **(D,E)** Similar to respective panels **A,B**, except for a spontaneous pain-like episode. Time 0 denotes the onset of paw withdrawal. **(F)** Receiver operating characteristic (ROC) curves of 5-fold cross-validated SVM classification for spontaneous pain. The AUROC and accuracy statistics (mean ± SEM) are shown in the inset.

During stimulus-evoked pain episodes, the spike activities of pain-modulated ACC and S1 units were temporally coordinated (by increasing or decreasing firing rates synchronously) to signal the pain onset (Figure 6A). Using an unsupervised population decoding algorithm (Methods), we identified the onset of evoked pain signals based on the ACC and S1 ensemble spike activity in single trials (Figure 6B). Among those successfully detected trials, the onset of detected acute pain signals matched or correlated closely with the ERP peak latency (Figure 6C, success ratio: 24/32 trials in session 3 from rat #15). In contrast, the change level in neuronal population spiking during spontaneous pain-like events was consistently lower, and the ERP peak amplitude was also considerably smaller (Figure 2G). Together, this posed a greater challenge for detecting the onset of spontaneous pain signals (Figures 6D,E).

To distinguish spontaneous pain-like episodes from pain-free negative control, we further trained a SVM classifier (Methods) using the combined LFP power features from the ACC and S1. Using 5-fold cross-validation on a total of 252 spontaneous pain episodes (rats #12–15, 10 sessions), we obtained a mean classification accuracy of 75% and an AUROC (area under the curve of receiver operating characteristic) of 0.85 (Figure 6F). By assessing the contribution of individual LFP power features, we found that the low-gamma and high-gamma power features from both the ACC and S1 were associated with more significant weights (Figure S8).

## Discussion

To date, most human or animal pain research has focused on stimulus-evoked pain, whereas spontaneous pain has not been fully investigated (Baliki et al., 2007; Bennett, 2012). In neuropathic pain, spontaneous pain is thought to emerge as a consequence of ectopic activity in axons in the injured nerve's action potentials arising spontaneously from hyper excitable membranes; but it remains unclear whether the source of this activity originates in injured neurons or in neighboring intact ones, and in nociceptors or non-nociceptors (Djouhri et al., 2006; Woolf, 2010). In contrast to human pain research (He et al., 2017), the lack of self-report has created a great obstacle for studying spontaneous pain in animal models (Tappe-Theodor and Kuner, 2014). A strong limitation and potential critique of our study is the subjective criterion for identifying spontaneous pain-like events. Although pain is usually inferred from stereotyped pain-like behaviors, other experimental techniques (such as spinal cord post-stimulation and nociceptive recording) can be used to help identify or confirm pain experiences. In the future, integrating behavioral with physiological measures may further help refine pain assessment for rodents (Whittaker and Howarth, 2014; Mouraux and Iannetti, 2018).

Simultaneous recordings of neuronal spikes and LFPs from multiple cortical areas provide a good opportunity to study differential neural mechanisms of evoked and spontaneous pain. In contrast to scalp or intracranial EEG signals, intracortical LFPs provide a more accurate readout from local microcircuits related to pain processing (Wang et al., 2015; Harris and Peng, 2016). Specifically, cortical ERP is a phase-locked signal generated by neuronal networks in relation to an externally or internally generated, yet behaviorally significant event. During evoked or spontaneous pain, ERPs are primarily contributed by the postsynaptic potentials of a large population of simultaneously active pyramidal cells with the same or similar orientation (Bressler, 2002). Our results show that ERPs from the ACC and S1 tend to synchronize during evoked pain episodes, but are highly variable during spontaneous pain-like episodes. While the ERP amplitudes are comparable between the ACC and S1, their amplitudes is greater during evoked pain than during spontaneous condition. In the frequency domain, the pain-evoked ERPs are directly linked to the low-frequency cortical oscillations (LeBlanc et al., 2014, 2017; Taesler and Rose, 2016; Peng et al., 2018). The theta-ERS has been demonstrated during evoked pain and spontaneous pain-like episodes, in either the ACC or S1, or both. Increased theta oscillations are possibly due to thalamic dysfunction or a decreased inhibition of the thalamus that affects pain processing (Stern et al., 2006). Previous studies have shown that increased theta power may represent a biomarker of chronic neuropathic pain (Pinheiro et al., 2016; Ploner et al., 2017).

The ACC and S1 are the two most important cortical regions related to pain processing. Neuroimaging studies in mice have shown that intra-regional remodeling within the S1 accelerates chronic pain behaviors by modulating the activity of ACC units (Eto et al., 2011). Specifically, the ACC displays cross-frequency coupling and spike-phase locking during pain perception in rats (Wang et al., 2011, 2015). Recent findings in rodent studies from our lab and others have suggested that ACC units are necessary for the “aversiveness” of pain (Johansen et al., 2001; Zhang et al., 2017). In addition, we have demonstrated that pairing auditory tones with repeated noxious stimulation can teach ACC neurons to produce a pain anticipation signal (Urien et al., 2018). Our rat LFP results of the ACC further support these previous findings that the ACC is a key component of an internal aversive network for both evoked and spontaneous pain. On the other hand, gamma oscillations in the human S1 have been shown to correlate with pain perception (Gross et al., 2007; Zhang et al., 2012; Tu et al., 2016), but these studies are limited to evoked pain. In the absence of overt noxious stimuli as in spontaneous pain episodes, the S1 may be involved in both the perception and modulation of various somatosensory sensations (Bushnell et al., 1999; Vierck et al., 2013). A rat S1 lesion study has implied a significant role of the S1 in pain affect without direct somatosensory processing, challenging the traditional view on the role of S1 in processing the sensory-discriminatory aspect of pain (Uhelski et al., 2012). Our rat S1 results during spontaneous pain-like episodes seem to support the lesion study. Nevertheless, a causal investigation (e.g., optogenetic S1 inactivation) is still required to fully dissect the role of the S1 in spontaneous pain perception. In some identified spontaneous pain-like episodes, we only observed ERPs in the ACC but not in the S1; this could be due to the fact that top-down input or anticipation was the primary driving force. However, further experimental investigation is required to distinguish between “spontaneous pain” and “pain anticipation.”

The functional state of cortical circuits may be defined by the amplitude and phase of ongoing frequency-specific oscillations of neuronal populations. LFP-based cross-frequency coupling measures nonlinear functional interactions between neural oscillations at different frequencies (Canolty and Knight, 2010; Tort et al., 2010). Complementary to amplitude-amplitude coupling (i.e., coherence), PAC may provide plausible physiological mechanisms on functional interactions—low-frequency phase reflects the local neuronal excitability, and high-frequency amplitude reflects the change in population synaptic activity or selective activation of a subnetwork in the microcircuit. To date, human pain studies have shown LFP cross-frequency coupling between the low-frequency phase (theta or alpha) and the gamma amplitude in the amygdala and hippocampus (Liu et al., 2015)—both regions are associated with pain and negative moods as a continuum of aversive behavioral learning (Baliki and Apkarian, 2015). Source-localized human EEG recordings have also shown theta phase-gamma amplitude coupling in the dorsal and subgenus ACC (Vanneste et al., 2018). One plausible interpretation of these findings is that theta oscillations reflect negative symptoms, and gamma oscillations obversely reflect positive symptoms. Theta oscillations may act as a traveling wave, communicating information across a large-scale network responsible for declarative or emotional memories (Zhang and Jacobs, 2015). On the other hand, gamma oscillations modulate long-range communication between distributed neuronal assemblies, which may subserve a wide range of cognitive functions including multi-sensory integration (Fries, 2009). Gamma oscillations can be nested on the theta wave for information transmission. While gamma oscillations may correlate with pain perception (Gross et al., 2007; Ploner et al., 2017), the high gamma activity can have a broader role in sensory processing. In addition, mechanisms of gamma sub-bands may have different origins, depending on differential distribution of cell types and cortical layers that receive thalamic or cortical input (Buzsaki and Wang, 2012). Our results have indeed showed that the strengths of PAC coupling vary across different gamma bands for the ACC and S1. A speculative neural coding role of PAC coupling is to segregate sensory (pain) responses into specific temporal windows within different cortical regions. However, circuit mechanisms of pain-associated slow and fast gamma oscillations are still incompletely understood.

Detection and identification of subjective pain signals for humans or animals has been an active research topic (Brown et al., 2011; Huang et al., 2013; Vijayakumar et al., 2017). While this problem has been well studied for evoked pain events in freely behaving rats (Chen et al., 2017; Hu et al., 2018; Xiao et al., 2018; Zhang et al., 2018), the challenge for detecting spontaneous pain events still remains. Our supervised learning results suggest that LFP power features from multiple brain regions may help detect spontaneous pain-like events (Figure 6). The future decoding strategy is to consider integrating the information from multi-regional LFP and ensemble spike activity to detect pain signals.

The results derived from our rodent study have important clinical implications. First, the ERP and LFP phenomena observed at the ACC and S1 circuit levels may be examined from human high-density EEG recordings combined with advanced source localization techniques. This would further allow us to investigate both evoked and spontaneous pain episodes in human subjects. Second, our algorithmic development and investigation on pain decoding also provide insight into detecting evoked and spontaneous pain events based on human EEG recordings (Huang et al., 2013).

In conclusion, our report has revealed differential coding roles between the S1 and ACC in pain processing, as well as point to distinct neural mechanisms between evoked pain and putative spontaneous pain at both LFP and cellular levels. These findings may suggest important circuit mechanisms that induce distinct pain perception or behaviors.

## Data Availability

All datasets generated for this study are available upon the request of corresponding authors. Software is publicly available at http://www.cn3lab.org/software.html.

## Ethics Statement

All experimental studies were performed in accordance with the National Institutes of Health (NIH) Guide for the Care and Use of Laboratory Animals to ensure minimal animal use and discomfort, and were approved by the New York University School of Medicine (NYUSOM) Institutional Animal Care and Use Committee (IACUC).

## Author Contributions

ZC and JW designed the research. ZX, EM, QZ, DR, and HZ collected the data. ZX, EM, PK, DR, RT, and LS analyzed the behavioral data. ZX and QH analyzed the neurophysiological data. ZC wrote the paper with contributing input from all other authors.

### Conflict of Interest Statement

The authors declare that the research was conducted in the absence of any commercial or financial relationships that could be construed as a potential conflict of interest.
