# Supplementary material for: Cortical Pain Processing in the Rat Anterior Cingulate Cortex and Primary Somatosensory Cortex
Source: Front Cell Neurosci. 2019 Apr 24;13:165. doi: 10.3389/fncel.2019.00165 (PMC6492531; doi:10.3389/fncel.2019.00165)
Supplement: Supplementary file 1 [file Data_Sheet_1.PDF]

Online Supplementary Material

Cortical Pain Processing in the Rat Anterior Cingulate Cortex and Primary Somatosensory Cortex

Zhengdong Xiao, Erik Martinez, Prathamesh M. Kulkarni, Qiaosheng Zhang, Qianning Hou, David Rosenberg, Robert Talay, Leor Shalot, Haocheng Zhou, Jing Wang & Zhe Sage Chen*

* Corresponding author (email: zhe.chen@nyulangone.org)

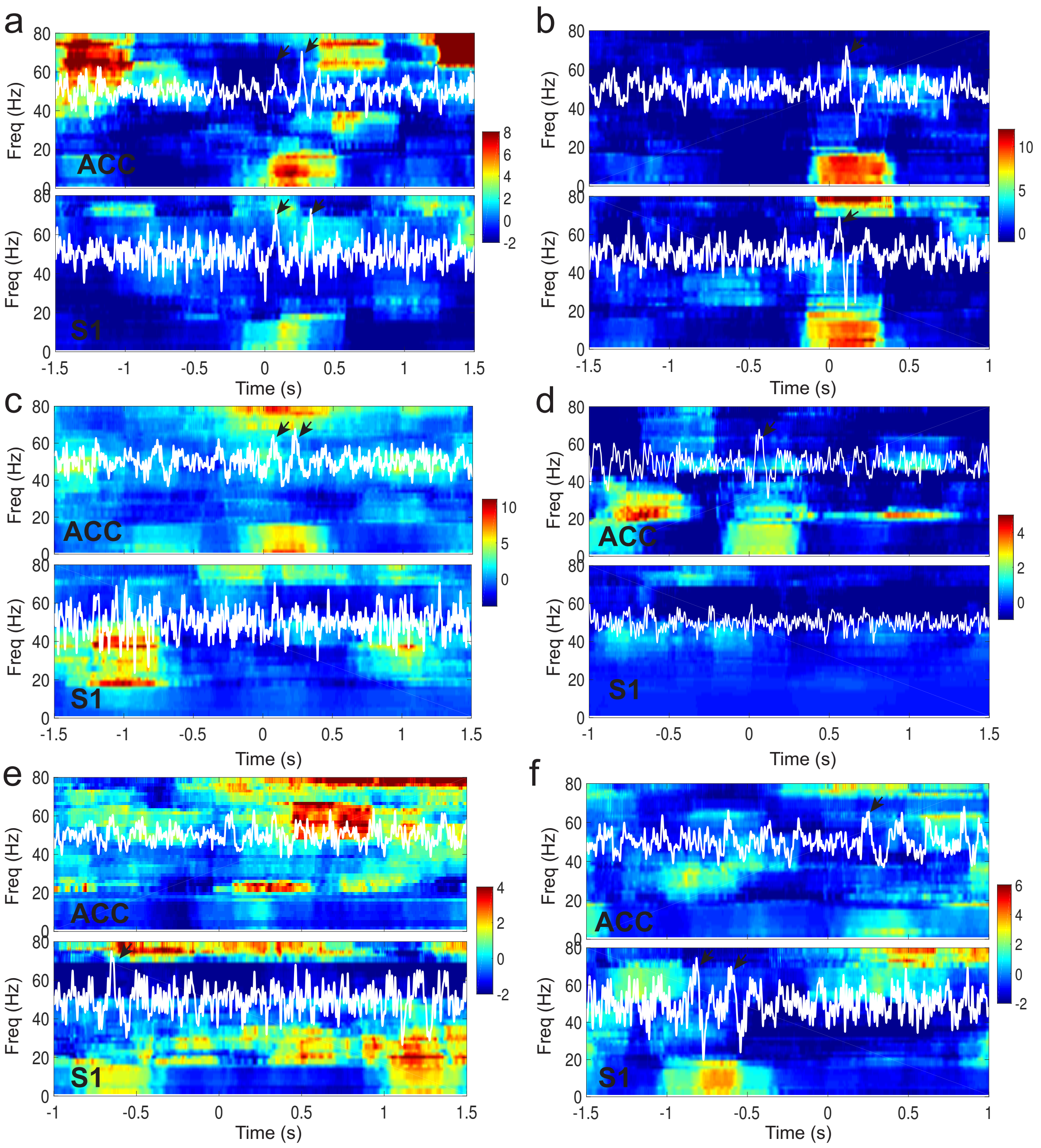

Figure S1: Examples of pain-induced ERPs and associated Z-scored spectrograms from the ACC and S1 during six spontaneous pain-like episodes. The power was Z-scored related to baseline (**a,b**) ERPs occurred nearly synchronously in the ACC and S1. (**c,d**) ERPs occurred in the ACC, but not in the S1. (**e**) ERPs occurred in the S1, but not in the ACC. (**f**) ERPs appeared first in the S1 then in the ACC.

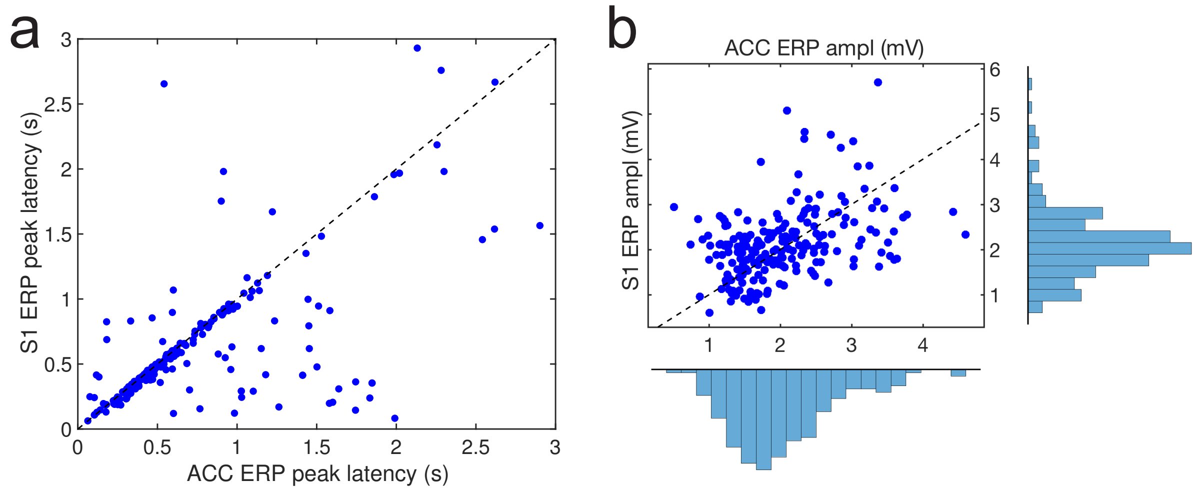

Figure S2: Comparison of ERP population statistics (ACC vs. S1) during evoked pain episodes ($n = 228$, rats #12-15). (**a**) The latency of ERP peak to the laser onset. The S1 ERP tended to appear earlier than the ACC ERP, with an averaged lag of 0.187±0.043 s. (**b**) The ERP peak amplitude. The ACC and S1 had comparable ERP amplitudes ($P > 0.05$, paired t-test).

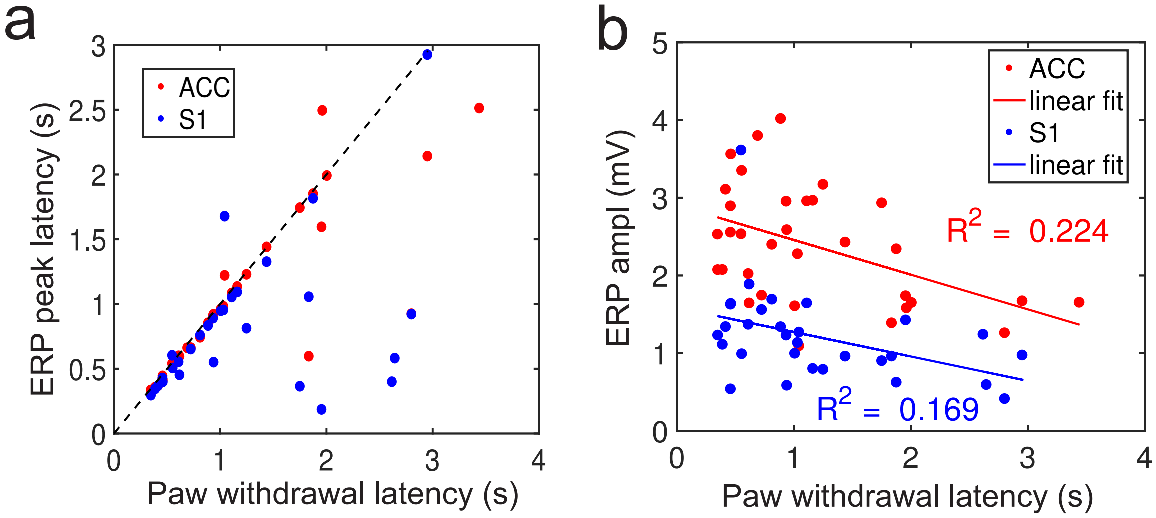

Figure S3: (**a**) ERP peak latency vs. paw withdrawal latency during evoked pain episodes. (**b**) The ACC or S1 ERP peak amplitude vs. paw withdrawal latency during evoked pain.

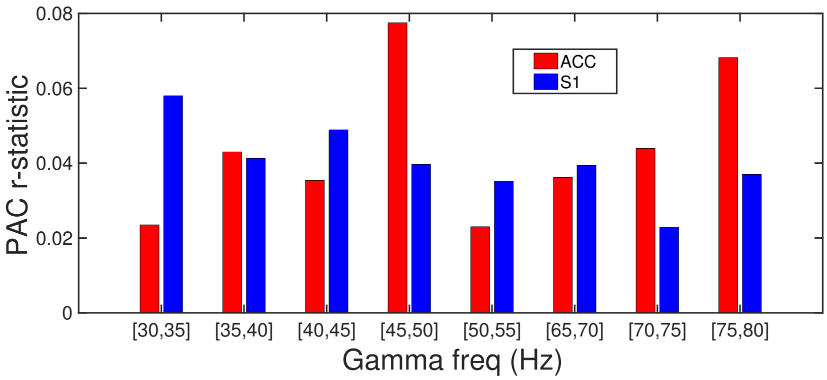

Figure S4: During evoked pain episodes, the strengths of PAC coupling of LFP signals varied according to gamma frequency sub-bands.

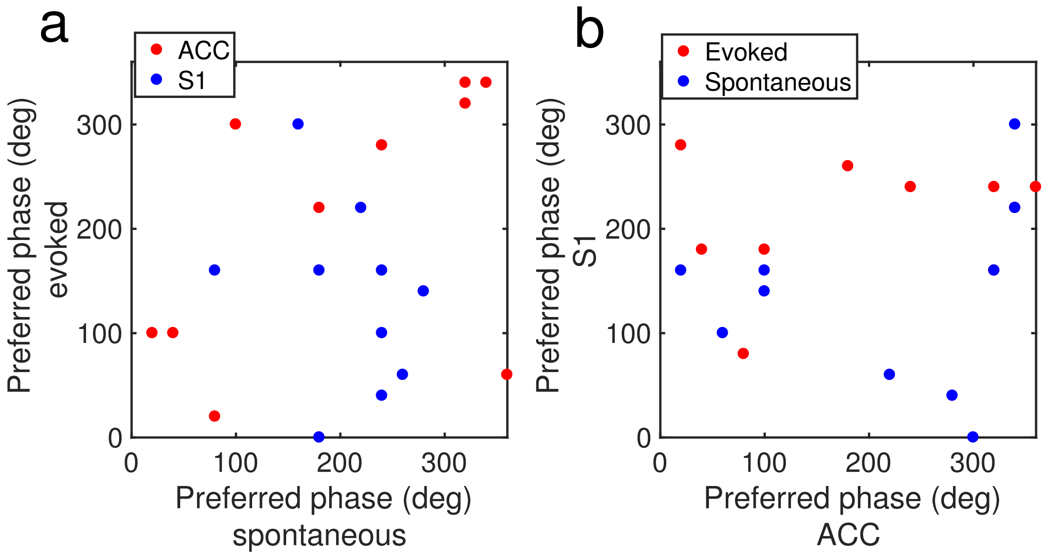

Figure S5: Comparison of the preferred theta phase in LFP theta-phase gamma-amplitude couplings between evoked pain and spontaneous pain-like episodes (**a**), as well as between the ACC and S1 (**b**). Rats #12-15, $n = 10$ sessions.

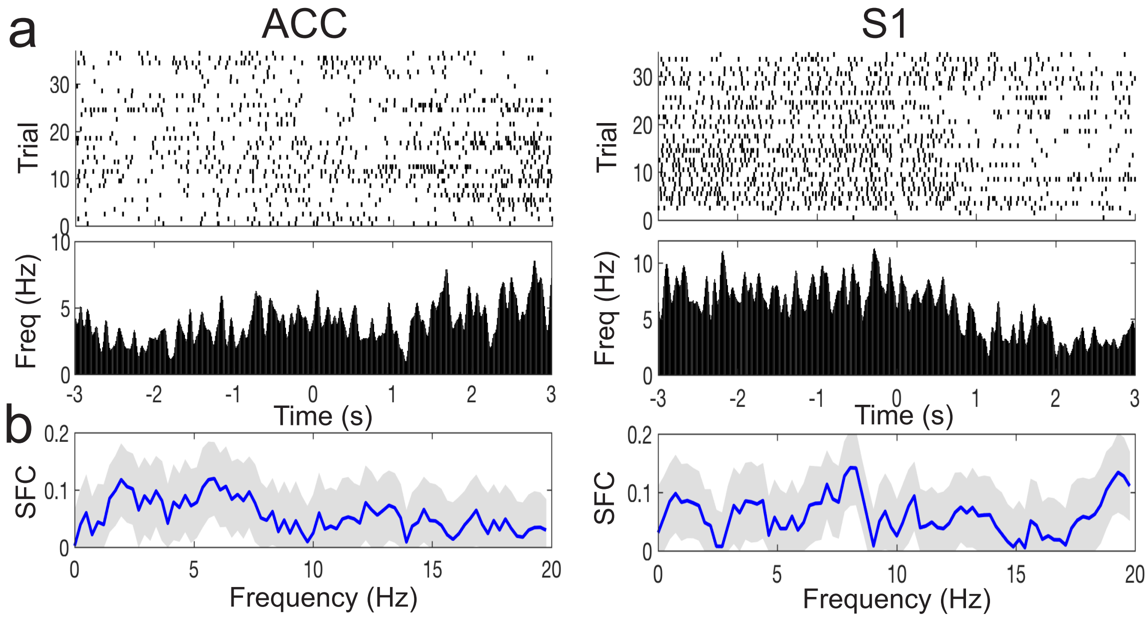

Figure S6: (**a**) Spike raster and PSTHs of one ACC unit and one S1 unit triggered on the stimulus onset (same spike train data as in Figure 5a and Figure 5g, respectively, but each trial was aligned on the laser onset). The PSTHs (bin size: 10 ms) appeared less sharper compared to the respective ones in Figure 5. (**b**) Trial-averaged spike-field coherence (SFC). Shaded areas denote the jackknife error bar. Notice the enhanced SFC in the theta band.

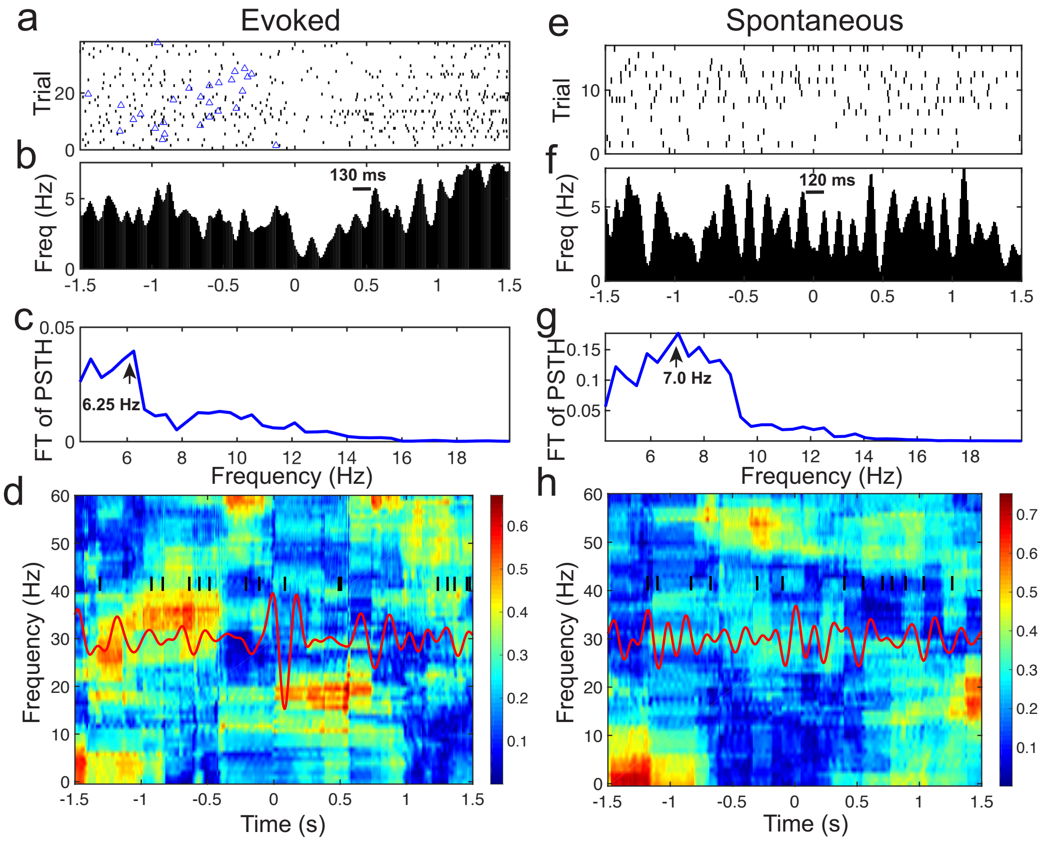

Figure S7: (**a**) Spike raster of a representative ACC unit during evoked pain episodes (same data as Figure 5a, but with a zoom-in view). Blue triangle at each row indicates the onset of noxious stimulus presentation at each trial. Time 0 represents the peak of ERP. (**b**) ERP-triggered PSTH (bin size: 10 ms). Note that "theta" oscillations were present in the single-unit spike activity. (**c**) Fourier transform (FT) magnitude of the PSTH in panel **b**. (**d**) SFC in a single-trial time-frequency representation (trial #11). Red trace represents the theta-band (4-8 Hz) filtered LFP signal in the ACC. Black ticks represents the ACC neuronal spike trains. Warm color represents high coherence. Starting around time 0, there was a strong SFC at the ACC beta frequency band. (**e-h**) Same as **a-d**, except for spontaneous pain-like episodes. Data in panel **e** are the same as the data in Figure 5d, but with a zoom-in view. Notice the striking similarity between these two conditions. In panels **d** and **h**, there was strong SFC at the ACC theta band around -1.5 s preceding the ERP.

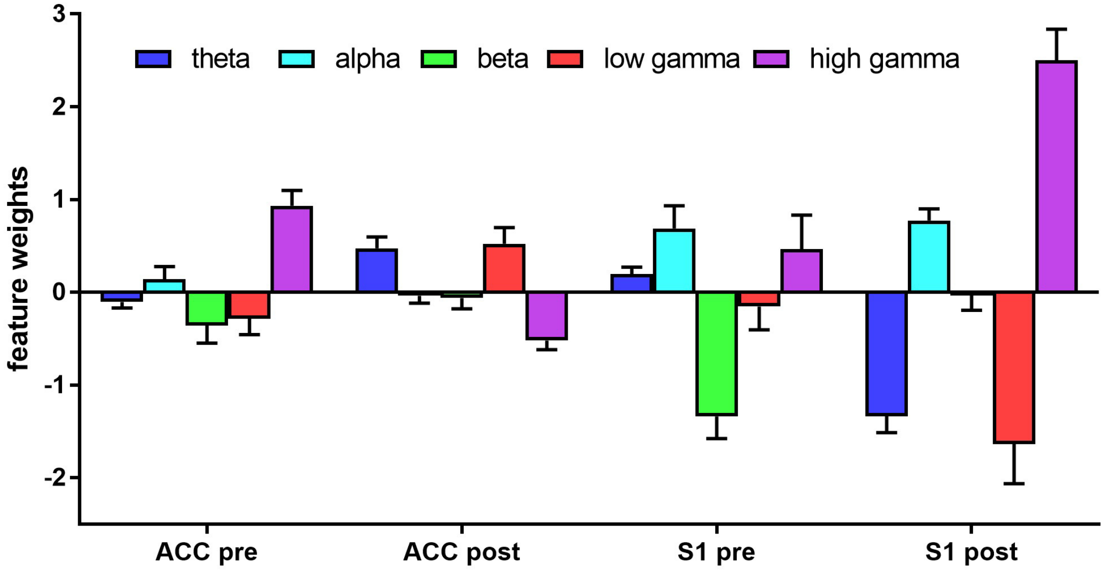

Figure S8: Feature weights associated with the LFP power features derived from the linear SVM classifier.
